# Effect of brand and advertising medium on demand for e-cigarettes: Evidence from an experimental auction^☆^

**DOI:** 10.1016/j.pmedr.2017.04.013

**Published:** 2017-04-27

**Authors:** Matthew C. Rousu, Richard O'Connor, Jay Corrigan

**Affiliations:** a Susquehanna University, 514 University Avenue, Selinsgrove, PA 17870, United States; b Roswell Park Cancer Institute, Elm & Carlton Streets, Buffalo, NY 14263, United States; c Kenyon College, 103 College Road, Gambier, OH 43022, United States

**Keywords:** Electronic cigarettes, Auctions, Experimental auctions, Cigarettes, Demand, Smoking, Willingness to pay

## Abstract

Print and television advertisements for e-cigarettes are currently legal in the United States. Given that e-cigarettes are a lower-risk alternative to cigarettes, these ads could have a positive public health impact if they motivate smokers to switch to e-cigarettes. However, the public health impact of e-cigarette ads could be negative if ads increase demand for both e-cigarettes and cigarettes. We use experimental auctions –in which participants bid in real auctions and winners pay for the items they purchase – to study the effect of print and TV e-cigarettes ads on demand for the brand from the ad, for another e-cigarettes brand, and for cigarettes. We ran experiments with 288 Pennsylvania smokers in November 2014–March 2015 and we found that in cases where an ad affects demand for e-cigarettes, the ad moves demand for cigarettes in the same direction. For example, the Blu print ad increases demand for Blu e-cigarettes and cigarettes among non-white participants. The Vuse TV ad reduces demand for both types of e-cigarettes and for cigarettes. We also find that non-white participants are willing to pay more for e-cigarettes in the absence of advertising, and that smokers who worry most about their health are willing to pay more for e-cigarettes. The results of this study point to the need for greater scrutiny of advertising for e-cigarette products such that they do not also induce demand for tobacco cigarettes.

## Highlights

• Print and television ads are currently legal in the United States.
• We use experimental auctions to assess how ads impact smokers demand for cigarettes and e-cigarettes.
• Examine ads for two major brands: Vuse and Blu
• Found that ads for e-cigarettes impact demand for cigarettes and e-cigarettes similarly

## 1 Introduction

Electronic cigarettes are part of an emerging class of products that deliver nicotine without combusting tobacco. Typically, this is accomplished through dissolving nicotine in a solution of propylene glycol and glycerin, and heating that solution to produce an aerosol that is inhaled by the user (El-Hellani et al., 2016, Breland et al., 2016). While such products are anticipated to be associated with substantial reduction of risks for smoking-associated diseases (McNeill et al., 2015), they remain controversial for several reasons, including renormalization of smoking behaviors (Cataldo et al., 2015), the use of flavorings attractive to youth (Goldenson et al., 2016), and unsubstantiated marketing claims (Klein et al., 2016). Sales of e-cigarettes have been rising steadily since 2012.

In April 2016, the Food and Drug Administration (FDA) released its final ‘deeming’ regulation, which extended its existing authorities over tobacco products to include e-cigarettes, hookah, cigars, and pipes (FDA, 2016). Among the regulations applied to e-cigarettes, which became effective in August 2016, is a requirement for manufacturers to register their products with FDA, include labels stating that the product contains nicotine (an addictive substance), and to limit sales to those aged 18 or over. Over the next two years, manufacturers will be required to produce data on the characteristics of their products, including contents and emissions of harmful and potentially harmful ingredients.

Understanding the dynamics of e-cigarette use, and in particular, smoker demand for such products, is important for projecting their likely impact on public health. If large numbers of smokers were to abandon cigarettes in favor of e-cigarettes, the anticipated effects could be large and positive. However, if most smokers simply use them as a situational substitute, the effects could be substantially smaller, and even negative if such use patterns depressed attempts to quit, or encouraged those who otherwise would not have used tobacco to take up e-cigarettes.

The same arguments apply to print and television advertisements for e-cigarettes, which are (as of this writing) legal in the United States. Intended use of e-cigarettes can be influenced by advertising, and e-cigarettes are unique in the US context in that advertising on television is currently permitted, a seeming advantage over other tobacco products. If these ads motivate smokers to switch from cigarettes to e-cigarettes, the public health impact is likely to be positive. But if e-cigarette ads increase demand for both cigarettes and e-cigarettes, the net public health effect may be negative.

Experimental economics offers methods that are designed to examine demand for products in a nonhypothetical way, which may provide important insights into how much smokers truly value e-cigarettes as an alternative to cigarettes. Experimental auctions, first developed in the 1960s, have been used by economists for over 20 years to assess the demand for many products, including irradiated food products (Fox et al., 2002), food labeled as genetically modified (Lusk, 2005), and organic foods (Akaichi et al., 2012). They also have been used more recently to assess issues in public health. This includes US smokers' demand for low and no-nicotine cigarettes (Rousu et al., 2005, Monchuk et al., 2007), smokeless tobacco (Rousu et al., 2014), cigarettes with graphic labels and plain packaging (Rousu and Thrasher, 2013, Thrasher et al., 2011), e-cigarettes (O'Connor et al., 2016), and to assess adult Mexican smokers' demand for cigarettes with pictorial vs. text-only warning labels (Thrasher et al., 2007). The current study was designed to estimate demand for cigarettes and two different types of e-cigarette brands as a function of print and TV e-cigarette advertisements.

## 2 Methods

### 2.1 Participant recruitment and sample size

The study protocol was approved by the IRB at Susquehanna University. Tables were set up at grocery stores in Shamokin, PA and Williamsport, PA between November 2014 and March 2015. Eligible study participants were 18 and older, had smoked > 100 cigarettes in their lifetimes, had smoked at least one cigarette in the last month, and were not pregnant. Posted signs indicated that adult smokers could earn $20 for 15–20 min of their time. Auctions were conducted with one to eight participants at a time, and a total of 288 subjects participated.

### 2.2 Experimental conditions

The study involved assessing how six advertising conditions along with a control group affected demand for two types of e-cigarettes and conventional cigarettes. Participants were randomly assigned to 1) a control group that saw no advertising; 2) a group given a print ad about Blu e-cigarettes; 3) a group given a TV ad about Blu e-cigarettes; 4) a group given both a print and a TV ad about Blu e-cigarettes; 5) a group given a print ad about Vuse e-cigarettes; 6) a group given a TV ad about Vuse e-cigarettes; and 7) a group given both a print and a TV ad about Vuse e-cigarettes. After surveying the companies' actual advertisements, we chose the ones we felt were most representative. Print ads can be found in the appendix while the Blu TV ad can be viewed at https://youtu.be/k79Iutkwdcg and the Vuse TV ad can be viewed at https://youtu.be/J4XpPXzg8J8. The TV ads were shown on an iPad while the print ads were laminated.

After seeing the ads (in all but the control group), participants placed bids. Participants bid on a single-use Blu e-cigarette, a single-use Vuse e-cigarette, and a pack of Camel cigarettes. For both types of e-cigarettes, participants bid on the type of e-cigarette they preferred: menthol or full flavor. For the Camel cigarettes, participants also bid on the type they preferred: they had the option to bid on full-flavored, light, menthol, or menthol-light.

### 2.3 Experimental design

For the current study, we used the Becker-DeGroot-Marschak (BDM) experimental auction mechanism (Becker et al., 1964). In this auction, participants are initially given enough money to compensate for their time and to provide them with more than enough money to pay the price that is randomly drawn for the product of interest. Each participant is allowed to examine the product and asked to place a bid on that product reflecting how much they would be willing to pay for it. Participants are told that this auction is different from other auctions in that they can only bid once and it is in their best interest to submit a bid equal to the full price they are willing to pay for the product. Further, participants don't pay the price of their own bid. After all bids are submitted, a price is selected randomly from a uniform distribution of prices. If a participant's bid is equal to or more than this randomly selected price, he or she purchases the product *paying the selected price*; a participant who bids less than the selected price does not purchase the product.

This BDM auction is “demand revealing” because it is theoretically in a participant's best interest to bid his or her true value (demand) for the product. This has been shown to hold in practice in the laboratory (Irwin et al., 1998, Alfnes et al., 2017) and the field (Corrigan and Rousu, 2008). That means we can confidentially interpret each participant's bid as his or her demand for the product. The demand revealing nature of this mechanism is why it has been used for hundreds of studies to estimate demand curves for products such as food labeled as genetically modified (Lusk et al., 2005), beef products with country of origin labels (Alfnes and Rickertson 2003), smokeless tobacco products (Rousu et al., 2014), nicotine free cigarettes (Monchuk et al., 2007), among many other products. (See Lusk and Shogren (2007) for a comprehensive overview.) This is in contrast with the more familiar first-price, sealed-bid auction, where the highest bidder wins the auction and pays a price equal to her bid. The first-price auction is not demand revealing because participants have an incentive to submit bids lower than their true value. Underbidding in a first-price auction can increase expected payoff because, while it reduces a participant's probability of winning the auction, it increases her payoff if she does win. Participants in a BDM mechanism have no incentive to understate their true value because the price auction winners pay is determined not by their bid, but rather a random draw. Someone who bids higher than her true value for the product could end up paying more than that true value, whereas someone who bids lower than her true value may miss out on a profitable purchase. Unlike surveys and focus groups, participants in experimental auctions make decisions that have a real and immediate financial impact (Lusk and Shogren, 2007, Huffman et al., 2003). In other words, auction winners pay for and receive the product, just as they would in the marketplace. This method offers the additional advantage of allowing greater experimental control over transaction conditions than studies of naturally occurring market transactions. For more on the properties of experimental auctions, see Lusk and Shogren (2007), Corrigan and Rousu (2008), or Alfnes and Rickertsen (2011).

### 2.4 Experimental protocol

After screening for eligibility and signing consent forms, participants filled out a brief survey on smoking behavior and received a detailed explanation of the BDM auction. Participants were informed (1) that they would place a private, written bid for an item; (2) they would purchase the product if their bid was higher than the bid randomly drawn from a container; and (3) it was in their best interest to bid their true value for the products, no more and no less. Participants were told that while they would bid on more than one product in a series of auction rounds, only one auction for one product would actually be carried out, and that that product would be randomly chosen after participants had bid on all products. This was done to disincentivize participants from reducing bids in later rounds in anticipation of winning multiple products. Any questions they had were answered and a practice round was conducted in which participants bid separately on two candy bars.

Participants then bid in the real auctions. The participants were presented with a single-use Blu e-cigarette and bid on it. Those bids were collected, and participants were then presented with a single-use Vuse e-cigarette and bid on it. Those bids were collected, and participants were presented with the pack of Camel cigarettes and bid on that pack. Once the three rounds of bidding were complete, the binding auction round (i.e., which product would be auctioned off) was randomly determined. The selected price was then randomly chosen from a uniform distribution, which ranged from $0.10 to $15.00 in increments of $0.10. If the participant bid equal to or more than this value, she paid the selected price and received the package. If the participant bid less, no purchase was made.

## 3 Analysis

To control for demographic and smoking-related characteristics, we estimate different Tobit regression models for cigarettes, Blu e-cigarettes, and Vuse e-cigarettes. A Tobit model corrects for the fact that bids are censored at zero. Bids for each product are the dependent variables. Independent variables include demographic variables, smoking-related variables, and dummy variables to indicate the advertising treatments (the no-advertising control group is the omitted category). The full regression model is estimated as.

*Bid*_*i*_ = *α*_0_ + *β*^′^*X*_*i*_ + *γ*^′^*S*_*i*_ + *δ*^′^*A*_*i*_ + *λ*^′^*N*_*i*_ + *ε*_*i*_,where *Bid*_*i*_ is participant *i*'s bid in for the product, *α*_*o*_ is an intercept term, *X*_*i*_ is a vector of demographic variables and *β*^′^ is the associated coefficient vector, *S*_*i*_ is a vector of smoking-related variables and *γ*^′^ is the associated coefficient vector, *A*_*i*_ is a vector of advertising treatment dummy variables and *δ*^′^ is the associated coefficient vector, *N*_*i*_ is a vector of advertising treatment dummy variables interacted with the “nonwhite or Hispanic” dummy variable and *λ*^′^ is the associated coefficient vector, and *ε*_*i*_ is the error term.

## 4 Results

The participants' average age was 39.6 years, and our sample was 56% female. Eighty-five percent of participants were white, and 60% had a household income of under $30,000 annually. Participants smoked an average of 18.5 cigarettes per day and 57% of participants had tried an e-cigarette. Table 1 contains unconditional results. Participants bid $4.54 for cigarettes, $6.41 for Blu e-cigarettes, and $9.12 for Vuse e-cigarettes on average. The differences in mean bids were statistically significant at the 1% level.

**Table 1:** Unconditional means (*N* = 288).

	
Bid for cigarettes	4.54 (1.99)^a^
Bid for Blu e-cigarettes	6.41 (5.61)
Bid for Vuse e-cigarettes	9.12 (9.49)
Age	39.62 (13.31)
Percent female	0.56
Percent white	0.85
Percent black	0.10
Percent Hispanic/Latino	0.06
Percent with income < 30 K	0.60
Percent with income between 30 K–60 K	0.20
Percent with income over 60 K	0.05
Percent income – declined to answer	0.15
Number of cigarettes daily	18.53
Never used e-cigarettes in the past	0.43
Highest education level is high school diploma or lower	0.72

Experiments conducted between November 2014 and March 2015.

a Standard deviations in parentheses.

Table 2 presents the results of the Tobit models used to examine demand for cigarettes and e-cigarettes. The models differ based on the dependent variable. Model 1 presents regression results where the dependent variable is the bid for cigarettes, Models 2 presents results where the dependent variable is the bid for Blu e-cigarettes, and Model 3 presents results where the dependent variable is the bid for Vuse e-cigarettes. The Vuse TV ad had a negative and statistically significant coefficient for two of the three models. White, non-Hispanic participants who saw the Vuse TV ad bid $0.94 less for cigarettes, and $2.18 less for Blu e-cigarettes. Relative to White, Non-white or Hispanic participants – which we combine because of a small sample size for either group separately – who saw the Blu print ad bid $2.85 more for cigarettes and $5.90 more for Blu e-cigarettes. And non-white or Hispanic participants who saw the Vuse TV ad bid $9.34 less for Vuse e-cigarettes relative to white participants.

**Table 2:** Censored regression model regressing bid for conventional cigarettes or e-cigarette. Standard error in parentheses. (*N* = 288).

	**1**	**2**	**3**
Variable	Cigarettes	Blu e-cigarettes	Vuse e-cigarettes
Intercept	4.97⁎⁎⁎ (0.60)	7.50⁎⁎⁎ (1.43)	10.38⁎⁎⁎ (2.68)
Treatment_bluPR	− 0.74⁎ (0.42)	− 1.07 (1.02)	− 2.99 (1.91)
Treatment_bluTV	− 0.61 (0.48)	− 0.88 (1.14)	− 0.09 (2.14)
Treatment_bluBoth	− 0.92 (0.60)	− 0.83 (1.45)	0.82 (2.71)
Treatment_VusePR	− 0.80 (0.80)	− 1.60 (1.92)	− 0.49 (3.59)
Treatment_VuseTV	− 0.94⁎⁎ (0.47)	− 2.18⁎ (1.14)	− 2.25 (2.12)
Treatment_VuseBoth	− 0.61 (0.48)	− 0.034 (1.15)	− 0.42 (2.15)
Female	− 0.31 (0.27)	0.32 (0.65)	1.41 (1.23)
NonWH_or_Hisp	− 0.20 (0.87)	0.66 (2.08)	9.46⁎⁎ (3.90)
Ed_HSorless	0.32 (0.29)	0.37 (0.70)	0.66 (1.31)
Income_Under30	0.47 (0.35)	0.19 (0.83)	− 0.18 (1.57)
Income_30_60	0.26 (0.42)	1.66 (1.01)	3.11 (1.90)
Age	− 0.01 (0.01)	− 0.03 (0.02)	− 0.05 (0.05)
EcigUSE_never	0.35 (0.26)	0.33 (0.62)	− 0.76 (1.17)
Worries	0.48 (0.31)	1.73⁎⁎ (0.73)	2.60 (1.37)
Number_cigs	0.00 (0.01)	− 0.06⁎ (0.03)	− 0.08 (0.06)
Cross_nonWH_bluPR	2.85⁎⁎ (1.29)	5.90⁎ (3.08)	5.93 (5.78)
Cross_nonWH_bluTV	0.49 (1.67)	− 0.05 (4.00)	− 8.96 (7.49)
Cross_nonWH_bluBoth	1.52 (1.23)	1.25 (2.94)	− 6.79 (5.51)
Cross_nonWH_vusePR	3.45 (2.28)	2.90 (5.46)	− 2.03 (10.22)
Cross_nonWH_vuseTV	− 0.04 (1.10)	− 0.35 (2.63)	− 9.34⁎ (4.92)
Cross_nonWH_vuseBoth	0.13 (1.15)	0.00 (2.75)	− 8.57 (5.16)

Experiments conducted between November 2014 and March 2015.

⁎⁎⁎ *p* < 0.01.

⁎⁎ *p* < 0.05.

⁎ *p* < 0.10.

Non-white or Hispanic participants who were not presented with ads bid $9.46 more for Vuse e-cigarettes, a difference that was statistically significant. Participants who worried about their health bid a statistically significant $1.73 more for Blu e-cigarettes. Number of cigarettes smoked had a marginally statistically significant $0.06 negative effect on bids for Blu e-cigarettes.

## 5 Discussion

E-cigarettes are another in a line of potentially reduced exposure products that have promised to reduce the risk of smoking. Our study examined smokers' preferences for two brands of e-cigarettes, Blu and Vuse, using an experimental auction. We found that smokers were willing to spend their money for e-cigarettes. In fact, given a single-use e-cigarette contains about two cigarette packs' worth of content, and that the bids for Vuse were double the bids for cigarettes, smokers had the same units/day bid for Vuse e-cigarettes as for cigarettes. At the same time, the units/day bid for Blu e-cigarettes was lower than for cigarettes. This difference in demand across brands reflects market conditions, as Vuse has recently overtaken Blu in terms of market share (Herzog et al., 2016). The Vuse product itself also has more attractive packaging (a transparent plastic box versus an opaque cardboard box for Blu), which may have contributed to a greater valuation by consumers.

We found that the Vuse TV ad decreased demand for cigarettes and Blu e-cigarettes among white, non-Hispanic smokers. One possible explanation is that smokers are cynical and turned off by pro-tobacco advertising. This would be consistent with evidence presented in Rousu et al., 2014, where pro-smokeless tobacco information did not affect demand for smokeless tobacco products but anti-smoking information did increase demand. We also found that the Blu print ad increased nonwhite or Hispanic participants' demand for *both* cigarettes and Blu e-cigarettes. We found modest evidence that the Vuse TV ad decreased demand for cigarettes among nonwhite or Hispanic participants. Taken together, these results suggest that e-cigarette ads shift demand for cigarettes and e-cigarettes in the same direction. If true, this could have serious public health implications given that print and TV ads for e-cigarettes are currently legal in the United States. If effective e-cigarette ads coincidentally increase demand for cigarettes in some populations, this may offset the public health benefits from some smokers adopting lower risk e-cigarettes.

Limitations of the current study include localization in North-central Pennsylvania, relatively low racial/ethnic diversity among participants, and limitation to two cartridge-based e-cigarette products. The latter limitation was driven by the need for paired print and TV advertising, since other forms of e-cigarettes (tank systems, eGo, Mods) are not advertised in this way, but rather by word-of-mouth, in vape shops, or over the Internet (Seidenberg et al., 2016). Future research should look to adapt and apply auction mechanisms to these emergent products, which have grown in popularity but whose use is harder to quantify given market diversity (Herzog et al., 2016, Zhu et al., 2014). We also only examined demand among current cigarette smokers. Demand for these products among nonsmokers, particularly youth, is also important to investigate. This is especially important given emerging evidence of nontrivial use levels. Another potential limitation is that our sample size might not have been large enough to capture differences across treatments.

E-cigarettes hold potential to reduce smoking-associated disease burden (McNeill et al., 2015). However, this rests on the presumption that they will attract large numbers of smokers away from cigarettes while not attracting substantial numbers of nonsmokers. Given the recent extension of FDA authority to regulate e-cigarettes, a mechanism now exists to investigate and enforce misleading advertising and unsupported claims. The results of this study point to the need for greater scrutiny of advertising for e-cigarette products such that they do not also induce demand for tobacco cigarettes.

## Figures and Tables

**Figure:**
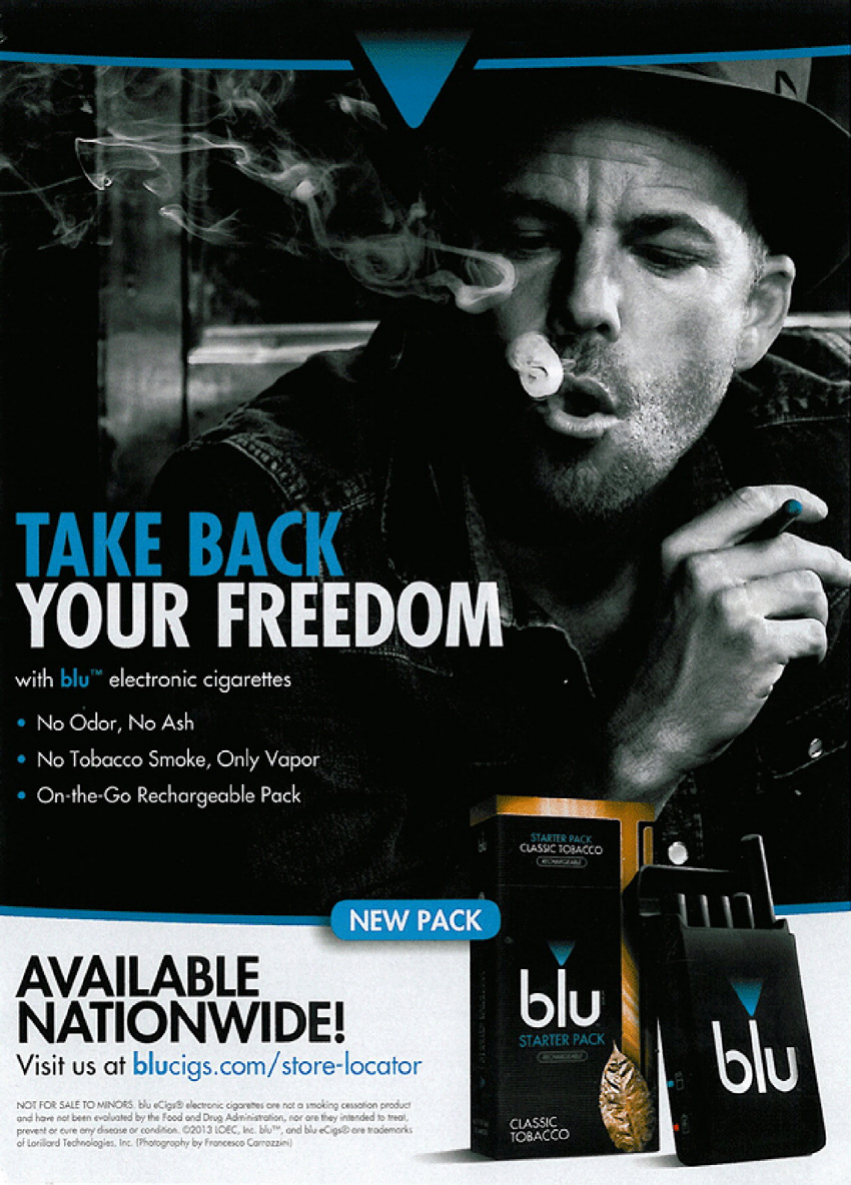


**Figure:**
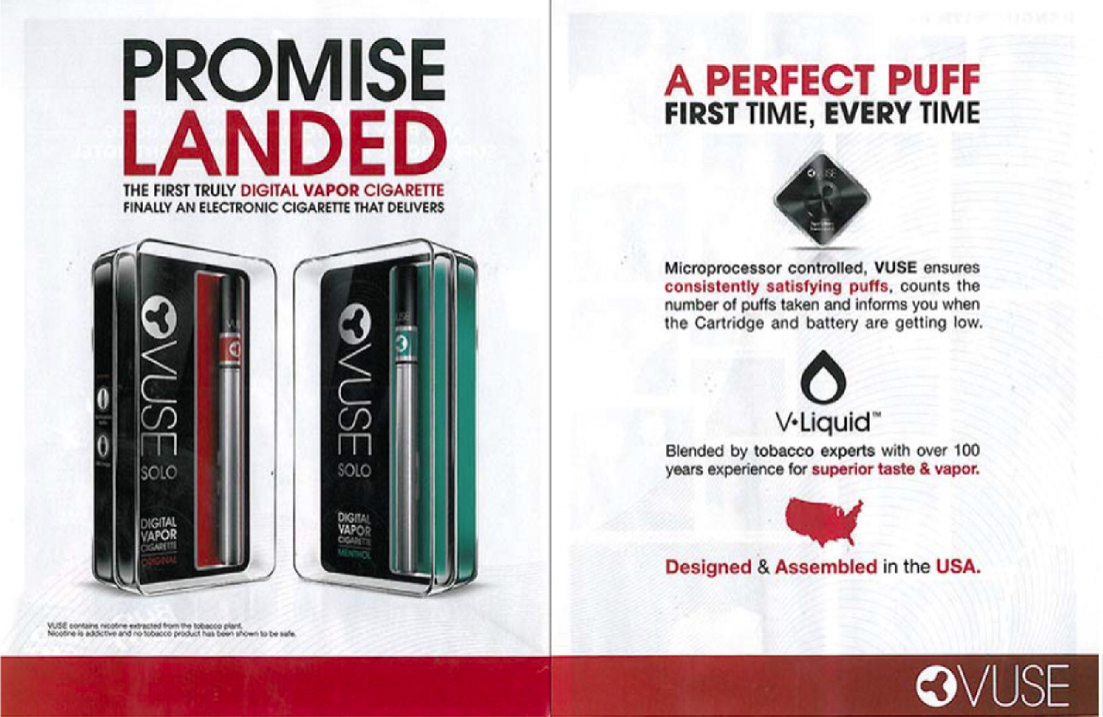


## References

[bb0005] Akaichi F., Nayga R.M., Gil J.M. (2012). Assessing consumers' willingness to pay for different units of organic milk: evidence from multiunit auctions. Can. J. Agric. Econ..

[bb9000] Alfnes F., Rickertsen K. (2003). European consumers' willingness to pay for US beef in experimental auction markets. Am. J. Agric. Econ..

[bb0010] Alfnes F., Rickertsen K., Lusk J.L., Roosen J., Shogren J.F. (2011). Non-Market Valuation: Experimental Methods. Oxford Handbook of the Economics of Food Consumption and Policy.

[bb0015] Alfnes F., Rickertsen K., Shogren J. (2017). Test-Retesting in Experimental Valuation of Perishable Food Products: Unstable Individual Bids and Reliable Market Demand.

[bb0020] Becker G.M., DeGroot M.H., Marschak J. (1964). Measuring utility by a single-response sequential method. Behav. Sci..

[bb0025] Breland A., Soule E., Lopez A., Ramôa C., El-Hellani A., Eissenberg T. (2016). Electronic cigarettes: what are they and what do they do?. Ann. N. Y. Acad. Sci..

[bb0030] Cataldo J.K., Petersen A.B., Hunter M., Wang J., Sheon N. (2015). E-cigarette marketing and older smokers: road to renormalization. Am. J. Health Behav..

[bb0035] Corrigan J.R., Rousu M.C. (2008). Testing whether field auction experiments are demand revealing in practice. J. Agric. Resour. Econ..

[bb0040] El-Hellani A., Salman R., El-Hage R. (2016). Nicotine and carbonyl emissions from popular electronic cigarette products: correlation to liquid composition and design characteristics. Nicotine Tob. Res..

[bb0045] Food and Drug Administration (FDA) (2016). Deeming tobacco products to be subject to the Federal Food, Drug, and Cosmetic Act, as amended by the family smoking prevention and tobacco control act; restrictions on the sale and distribution of tobacco products and required warning statements for tobacco products. Federal Reserve.

[bb0050] Fox J.A., Hayes D.J., Shogren J.F. (2002). Consumer preferences for food irradiation: how favorable and unfavorable descriptions affect preferences for irradiated pork in experimental auctions. J. Risk Uncertain..

[bb0055] Goldenson N.I., Kirkpatrick M.G., Barrington-Trimis J.L. (2016). Effects of sweet flavorings and nicotine on the appeal and sensory properties of e-cigarettes among young adult vapers: application of a novel methodology. Drug Alcohol Depend..

[bb0060] Herzog B., Kanada P., Scott A. (2016). Nielsen: Tobacco “All Channel” Data Through 10/08: Measured Channel Check Confirms Deceleration in Cig Volume Trends.

[bb0065] Huffman W.E., Shogren J.F., Rousu M., Tegene A. (2003). Consumer willingness to pay for genetically modified food labels in a market with diverse information: evidence from experimental auctions. J. Agric. Resour. Econ..

[bb0070] Irwin J.R., McClelland G.H., McKee M., Schulze W.D., Norden N.E. (1998). Payoff dominance vs. cognitive transparency in decision making. Econ. Inq..

[bb0075] Klein E.G., Berman M., Hemmerich N., Carlson C., Htut S., Slater M. (2016). Online E-cigarette marketing claims: a systematic content and legal analysis. Tobacco Regulatory Science.

[bb0080] Lusk J.L. (2005). Consumer welfare effects of introducing and labeling genetically modified food. Econ. Lett..

[bb0085] Lusk J.L., Shogren J.F. (2007). Experimental Auctions: Methods and Applications in Economic and Marketing Research.

[bb9005] Lusk J.L., Jamal M., Kurlander L., Roucan M., Taulman L. (2005). A meta-analysis of genetically modified food valuation studies. J. Agric. Resour. Econ..

[bb0090] McNeill A., Brose L.S., Calder R., Hitchman S.C., Hajek P., McRobbie H. (2015). E-cigarettes: An Evidence Update – A Report Commissioned by Public Health England.

[bb0095] Monchuk D.C., Rousu M.C., Shogren J.F., Nonnemaker J., Kosa K.M. (2007). Decomposing the value of cigarettes using experimental auctions. Nicotine Tob. Res..

[bb0100] O'Connor R.J., Rousu M.C., Bansal-Travers M., Vogl L., Corrigan J.R. (2016). Using experimental Auctions to examine demand for E-cigarettes. Nicotine Tob. Res..

[bb0105] Rousu M.C., Thrasher J.F. (2013). Demand reduction from plain and pictorial cigarette warning labels: evidence from experimental auctions. Appl. Econ. Perspect. Policy.

[bb0110] Rousu M.C., Monchuk D.C., Shogren J.F., Kosa K.M. (2005). Consumer willingness to pay for “second-generation” genetically engineered products and the role of marketing information. J. Agric. Appl. Econ..

[bb0115] Rousu M.C., O'Connor R.J., Thrasher J.F., June K.M., Bansal-Travers M. (2014). The impact of product information and trials on demand for smokeless tobacco and cigarettes: evidence from experimental auctions. Prev. Med..

[bb0120] Seidenberg A.B., Jo C.L., Ribisl K.M. (2016). Differences in the design and sale of e-cigarettes by cigarette manufacturers and non-cigarette manufacturers in the USA. Tob. Control..

[bb0125] Thrasher J.F., Rousu M.C., Anaya-Ocampo R., Reynales-Shigematsu L.M., Arillo-Santillán E., Hernández-Ávila M. (2007). Estimating the impact of different cigarette package warning label policies: the auction method. Addict. Behav..

[bb0130] Thrasher J.F., Rousu M.C., Hammond D., Navarro A., Corrigan J.R. (2011). Estimating the impact of pictorial health warnings and “plain” cigarette packaging: evidence from experimental auctions among adult smokers in the United States. Health Policy.

[bb0135] Zhu S.H., Sun J.Y., Bonnevie E. (2014). Four hundred and sixty brands of e-cigarettes and counting: implications for product regulation. Tob. Control..

